# Omega-3 fatty acids modulate acetamiprid and emamectin benzoate-induced testicular toxicity in rats by modulating Nrf2/NFkB pathway and apoptotic signaling

**DOI:** 10.1038/s41598-025-30696-w

**Published:** 2025-12-17

**Authors:** Fatma Mohamady El-Demerdash, Mohamed Yousef Fayed, Ehab Moustafa Tousson, Raghda Ahmed El-Sayed

**Affiliations:** 1 Department of Environmental Studies, Institute of Graduate Studies and Research, Alexandria University, 163 Horreya Avenue, P.O. Box 832, Alexandria, 21526 Egypt; 2 Department of Zoology, Faculty of Science, Tanta University, Tanta, 31527 Egypt

**Keywords:** Acetamiprid/emamectin benzoate, Omega-3 fatty acids, Oxidative stress, Biochemical/molecular analysis, Histology/immunohistochemistry, Testicular dysfunction, Biochemistry, Physiology, Zoology

## Abstract

Pesticides are well-known harmful substances that cause oxidative stress and testicular dysfunction in both humans and animals, whereas omega-3 fatty acids (ω3FAs) have been demonstrated to possess antioxidant and anti-inflammatory properties. Thus, the primary focus of this investigation was the protective role of (ω3FAs) and their related molecular mechanism in testicular dysfunction induced by acetamiprid plus emamectin benzoate in rats. Rats were divided into four groups: control, omega-3 fatty acids (ω3FA; 300 mg/kg BW), insecticide mixture (Insec Mix; Acetamiprid (30 mg/kg BW) and emamectin benzoate (9 mg/kg BW)), and ω3FA + Insec Mix, respectively. ω3FA was taken orally an hour before insecticide treatment for three weeks daily. The results demonstrated that lipid peroxidation markers and lactate dehydrogenase activity were significantly elevated in rats intoxicated with pesticides; however, enzymatic antioxidants, aminotransferases, phosphatases, and reduced glutathione decreased. Furthermore, notable changes in testicular Bax, Cas-3, Bcl-2, P53, IL-1β, TNF-α, NFkB, Nrf2, hormones, sperm quality, testis structure, and Ki-67 protein expression were detected. Otherwise, ω3FA pre-treatment before insecticide intoxication substantially recovered most of the molecular and biochemical indicators and improved testicular cellular structure. Conclusively, ω3FA was highly effective in mitigating testicular toxicity conferred by acetamiprid and emamectin benzoate insecticides.

## Introduction

 Pesticides have greatly benefited humans by enhancing agricultural products and aiding in the prevention of infectious diseases. The neurological, endocrine, immunological, reproductive, renal, cardiovascular, and respiratory systems are just a few of the organs in the body that might be negatively impacted by their widespread usage, which could be dangerous for human health^1–5^.

Among the widely used insecticides are emamectin benzoate and neonicotinoids. Emamectin benzoate (EMB) is a macrocyclic lactone insecticide derived from the fermentation of *Streptomyces avermitilis*. It acts as a potent neurotoxic agent by disrupting neurotransmitter activity, specifically by activating glutamate-gated chloride channels, leading to paralysis and death in target insects^6^. Residues of EMB have been detected in surface water, topsoil, and agricultural products^7^. Neonicotinoids are regarded as one of the most significant chemical classes of insecticides brought to the worldwide market since synthetic pyrethroids. They are widely utilized for purposes related to animal health and crop protection. Subchronic exposure to EMB has been shown to impair germ cell maturation in the testes, leading to increased DNA fragmentation, lipid peroxidation, and depletion of antioxidant defense systems^6^. Acetamiprid (ACP) belongs to the class of neonicotinoid insecticides, which is preferred for its broad-spectrum activity and excellent efficacy in controlling a variety of insects^8,9^. However, due to its high-water solubility and long half-life, it tends to accumulate in the environment, leading to potential human exposure through contaminated food and water^8^. While initially considered to have lower mammalian toxicity, recent studies have demonstrated that sub-acute exposure to ACP induces significant oxidative stress and histopathological damage in the male reproductive system, characterized by increased lipid peroxidation and decreased total antioxidant capacity^8,10^. Generally, the main mechanism proposed to explain the cyto-genotoxicity of these insecticides is the generation of reactive oxygen species (ROS) resulting in oxidative injury^3,11^.

Consumption of food has a significant impact on controlling some degenerative processes that affect an organism’s quality of life. Antioxidants can scavenge free radicals and cleanse the body, preventing their detrimental effects by slowing down or stopping the oxidation of lipids or other molecules^12,13^. Omega-3 polyunsaturated fatty acids are beneficial to health, particularly in their long-chain forms, docosahexaenoic acid (DHA) and eicosapentaenoic acid (EPA)^14^. ω3FA is frequently present in oils from cold-water fish, like tuna and salmon. Additionally, they are present as α-linolenic acid in nuts, oils, and certain green vegetables. Their positive effects may be linked to their ability to act as antioxidants, lower levels of pro-inflammatory cytokines, and immune system protection^15–17^. Therefore, the current investigation aimed to explore the role of ω3FA in alleviating testicular dysfunction and toxic effects induced by acetamiprid and emamectin benzoate in rats.

## Materials and methods

### Materials

Omega-3 fatty acids (ω3FA) from fish oil were purchased from Sigma Chemical Company (USA). Emamectin benzoate (EB (T 20180803)) (4́-deoxy-4́-epimethylamino-4́-deoxyavermectin B1 benzoate, (> 95% pure) is a product that was purchased from Hebei Veyong Bio-Chemical Co., Ltd. (Shijiazhuang, China). Acetamiprid (E)-N-[(6-chloro-3-pyridinyl) methyl]-N´- cyano-N- methylethanimidamide, C_10_H_11_C_l_N_4_) (> 97% pure) was purchased from Shanghai Yongyuan Chem. Ltd. (Shanghai, China).

### Experimental plan

The animal house of the Faculty of Medicine, Alexandria University, Alexandria, Egypt, provided 24 male Wistar Albino rats, which weighed between 150 and 170 g. The experimental plan was accepted by the Local Ethics Committee and Animals Research of Alexandria University (#AU14-240922-3-4), and the protocol complies with the requirements of the ARRIVE Guidelines (https://arriveguidelines.org) for the care and use of Laboratory animals. The rats were housed in wire cages with stainless steel bottoms, maintained at standard conditions (22 ± 2 °C temperature, 40–60% relative humidity, and a 12-hour light/dark cycle), and provided with free access to water and a pellet diet. Rats were acclimated for two weeks before being divided randomly into 4 groups (6 rats/group). Rats were treated as follows: group 1 (control) was given corn oil (0.5 ml/kg BW), group II was administered omega 3-fatty acids (ω3FA; 300 mg/kg BW), group III was treated with insecticide mixture (Insect Mix; Acetamiprid 30 mg/kg BW, $\:\cong\:$1/7 LD_50_, oral rats LD_50_ 200 mg/kg) and emamectin benzoate (9 mg/kg BW, $\:\cong\:$1/10 LD_50_; oral rats LD_50_ 89 mg/kg), and group IV was given ω3FA an hour before Insec Mix treatment, respectively. Doses of ω3FA^18^, acetamiprid^8^, and emamectin benzoate^7,19^were administered orally by gavage every day for three weeks. Insecticide-treated rats showed no evidence of morbidity or death. Rats were starved overnight on the last day of the experiment. Ultimately, rats from each group were euthanized after being isoflurane-anesthetized, and then blood and testis were collected for further examinations.

### Serum and tissue preparation

Blood samples obtained from heart punctures were centrifuged for 15 min at 2000xg after being left to clot for 30 min at 25 °C. The transparent sera were then meticulously isolated and kept at −20 °C for additional research. To prepare tissue homogenate, testes were removed from the animals after being dissected and then homogenized (10% w/v) in cold 0.01 mol/L sodium-potassium phosphate buffer pH 7.4, and centrifuged at 10,000 xg for 20 min (4 °C), and the clear supernatants were preserved at −20 °C for further analysis.

### Evaluation of lipid peroxidation, enzymatic and non-enzymatic antioxidants

Lipid peroxidation was measured in rats’ testicular homogenates by detecting the levels of thiobarbituric acid reactive substances (TBARS)^20^ and hydrogen peroxide (H_2_O_2_)^21^. The activities of superoxide dismutase (SOD; EC 1.15.1.1) and catalase (CAT; EC 1.11.1.6) were evaluated according to Alam et al.^22^ and Raghunathan et al.^23^, respectively. The activities of glutathione peroxidase (GPx; EC 1.1.1.9) and glutathione reductase (GR; EC 1.8.1.7) were examined by Sood and Singla^24^. The activity of glutathione S-transferase (GST; EC 2.5.1.18) was assayed according to Habig et al.^25^, while reduced glutathione (GSH) content was analyzed by Davies et al.^26^.

### Determination of biochemical parameters

Aspartate aminotransferase (AST; EC 2.6.1.1, CAT.NO. AS 10 61), alanine aminotransferase (ALT; EC 2.6.1.2, CAT.NO. AL 10 31), lactate dehydrogenase (LDH; EC 1.1.1.27; CAT NO. 117125), alkaline phosphatase (ALP; EC 3.1.3.1, CAT. NO. AP 10 20), and acid phosphatase (AcP; EC 3.1.3.2, CAT. NO. AC 10 10) activities, as well as protein content (CAT. NO. TP 20 20) were assessed spectrophotometrically by commercially available kits obtained from Biodiagnostic Company, Egypt.

### *S*perm parameters

The left caudal epididymis of each testicle was delicately removed, chopped in Hank’s solution for free spermatozoa movement, and then stored at 25 °C for 15 min. A light microscopic examination (Olympus, Tokyo, Japan) and computer-assisted semen analysis were used to assess the sperm properties^27,28^.

### Analysis of hormones

To measure the concentrations of serum testosterone (T), luteinizing hormone (LH), and follicle-stimulating hormone (FSH), kits were used (RIA TESTO CTC KIT, Dia-Sorin Company: Stillwater, Minnesota, USA; RIA kits from NIADDK; Bethesda, MD, USA, and Elisa Kit, DiaMetra kits, Via Giustozzi, Italy), respectively.

### Molecular investigations

Using Glyceraldehyde-3-phosphate dehydrogenase (GAPDH) as an internal reference, the target gene mRNA expression in the testes was assessed using real-time PCR with SYBR Green. Gene-specific primers and 2X Maxima SYBR Green/ROX qPCR Master Mix were used to amplify the extracted cDNA, according to the manufacturer’s instructions (Thermo Scientific, USA, # K0221). Table 1 lists primer names and sequences used for RT-qPCR in this investigation. The theoretical 2^−∆∆CT^ formula was used for calculations^29^.

**Table 1:** The names and sequences of primers used in this study.

Gene	Forward primer	Reverse primer
**Apoptosis**		
*Bax*	ACACCTGAGCTGACCTTG	AGCCCATGATGGTTCTGATC
*Caspase3*	GGTATTGAGACAGACAGTGG	CATGGGATCTGTTTCTTTGC
*Bcl2*	AGTACCTGAACCGGCATCTG	CATGCTGGGGCCATATAGTT
*P53*	AACTGGAAGAATTCGCGGCCGCAGGAAT	GCTACCCGAAGACCAAGAAGG
**Inflammation**		
*IL1β*	CACCTCTCAAGCAGAGCACAG	GGGTTCCATGGTGAAGTCAAC
*TNF-α*	GCATGATCCGCGACGTGGAA	AGATCCATGCCGTTGGCCAG
*Nrf2*	TTGTAGATGACCATGAGTCGC	TGTCCTGCTGTATGCTGCTT
*NFkB*	CCTAGCTTTCTCTGAACTGCAAA	GGGTCAGAGGCCAATAGAGA
**Housekeeping**		
*GAPDH*	GTTACCAGGGCTGCCTTCTC	GGGTTTCCCGTTGATGACC

### Histopathological studies

The testes tissues were preserved for two days in neutral formalin buffer (10%), then placed in different alcohol concentrations for dehydration and embedded in paraffin. A light microscope (Olympus BX41, Japan) was used to photograph the paraffin slices after they had been stained with hematoxylin and eosin^30^. Mean seminiferous tubular and luminal diameter and epithelial height were estimated as reported earlier^31–33^. Mean Seminiferous Tubular Diameter (MSTD) of each testis was determined by measuring 20 separate round seminiferous tubules with a light microscope-adaptable micrometer. The mean of the values obtained was regarded as the MSTD of the testis.

### Immunohistochemistry

According to Tousson et al.^34^, Ki-67 immunohistochemistry was performed on 5 μm paraffin-embedded testicular tissue sections to evaluate cell proliferation. The sections were deparaffinized, rehydrated, and treated to block endogenous peroxidase activity. After washing with phosphate-buffered saline (PBS) and applying a blocking solution, the tissues were incubated with a mouse monoclonal Ki-67 antibody (1:100 dilution; DAKO Japan Co., Tokyo, Japan). This was followed by incubation with a biotinylated secondary antibody and a streptavidin–peroxidase complex. The reaction was visualized using diaminobenzidine (DAB) as the chromogen, which produced a brown coloration at antigen sites. The slides were then counterstained with hematoxylin, dehydrated, cleared, and mounted. Finally, stained sections were examined under an Olympus microscope, and digital images were captured and adjusted for brightness and contrast using Adobe Photoshop.

### Statistical analysis

Data from different groups were analyzed using SPSS software (version 22, IBM Co., Armonk, NY), and the results were presented as means ± standard errors (SEM). Tukey’s post-hoc test and ANOVA were used to compare groups. Statistically significant results were deemed valid at *P* < 0.05.

## Results

### Lipid peroxidation biomarkers

**Fig. 1:**
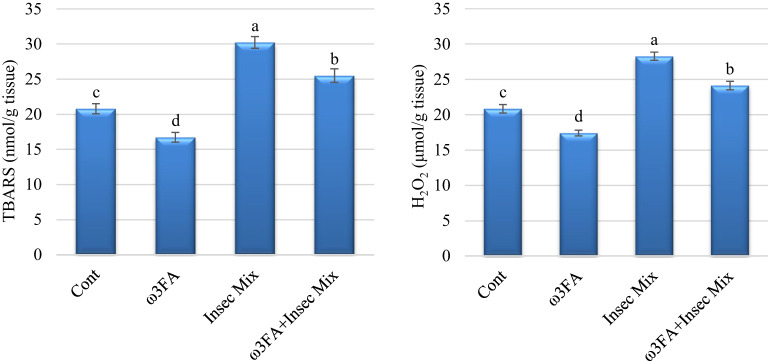
Lipid peroxidation markers in rat testes. Results are presented as means ± SE (*n* = 6/group). Various letters (a, b,c, and d) were used to convey significance (*P* < 0.05) across groups. ω3FA and Insec Mix groups are contrasted to the negative control group, while the ω3FA plus Insec Mix group is related to the positive control group, Insec Mix.

Male rats subjected to a combination of acetamiprid (ACP) and emamectin benzoate (EMB) exhibited a substantial rise in testes TBARS and H_2_O_2_ compared to the control group. Conversely, rats administered ω3FA alone demonstrated a noteworthy reduction in testes TBARS and H_2_O_2_. Lipid peroxidation indicators improved in rats treated with omega-3 fatty acids and Insec Mix when compared to Insec Mix (Fig. 1).

### Enzymatic and non-enzymatic antioxidants

**Fig. 2:**
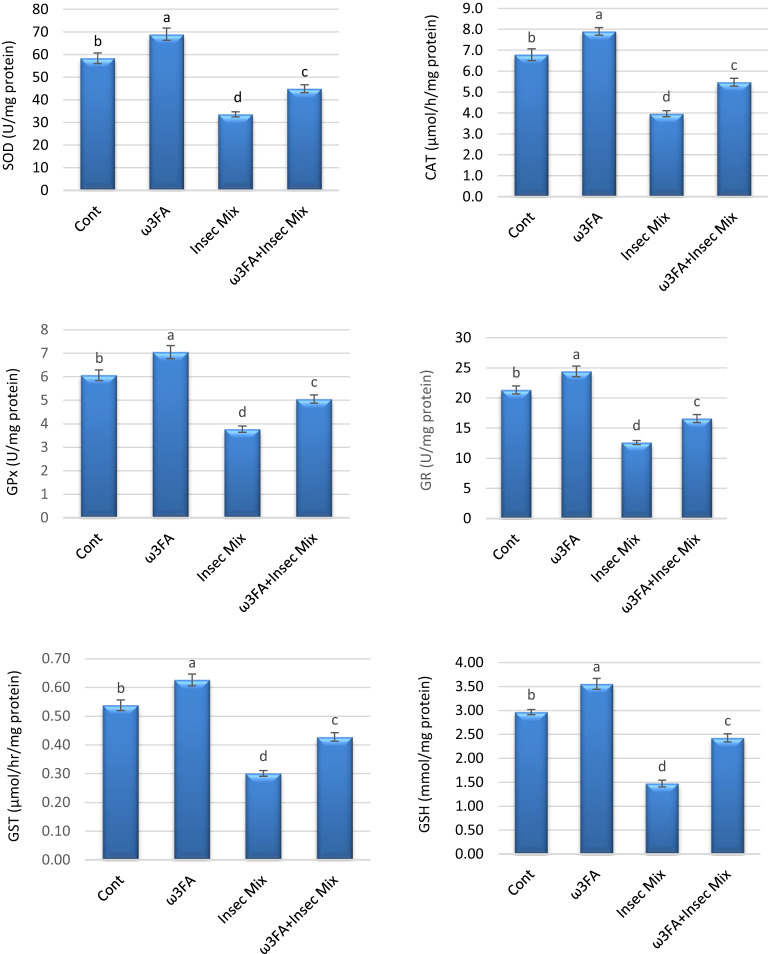
Enzymatic and non-enzymatic antioxidants in rat testes. Results are presented as means ± SE (*n* = 6/group). Various letters (a, b,c, and d) were used to convey significance (*P* < 0.05) across groups. ω3FA and Insec Mix groups are contrasted to the negative control group, while the ω3FA plus Insec Mix group is related to the positive control group, Insec Mix.

Male rats exposed to insecticides had significantly lower reduced glutathione (GSH) concentrations and lower levels of antioxidant enzymes (SOD, CAT, GPx, GR, and GST) compared to control rats. Contrariwise, a noteworthy rise in GSH, SOD, CAT, GPx, GR, and GST was detected in rats administered ω3FA alone, compared to controls. Moreover, the ω3FA + Insec Mix group revealed a substantial amendment in these indicators compared to the Insec Mix-treated group (Fig. 2).

### Enzyme activity and protein content

Male rats exposed to insecticides exhibited a considerable decrease in the activities of AST, ALT, ALP, ACP, and protein content; however, LDH activity increased compared to the control rats. In contrast, rats given ω3FA alone showed a non-significant change in these forementioned parameters related to the control ones. Rats administered omega-3 fatty acids before insecticides showed significant improvement in their testes’ enzymes and protein content compared to rats treated with insecticides alone (Table 2).

**Table 2:** Effect of omega-3 fatty acid (ω3FA), insecticides mixture (Insec Mix), and their combination (ω3FA + Insec Mix) on biochemical parameters.

Parameters	Groups			
	Cont.	ω3FA	Insec Mix	ω3FA + Insec Mix
AST (U/mg protein)	113 ± 3.76^b^	116 ± 3.66^a^ (+ 2.83)	84 ± 1.44^d^ (−25.08)	82 ± 2.85^c^ (−12.71)
ALT (U/mg protein)	213 ± 7.55^a^	223 ± 8.09^a^ (+ 4.35)	148 ± 5.18^c^ (−30.62)	177 ± 4.79^b^ (−17.16)
LDH (U/mg protein)	838 ± 22.05^c^	786 ± 31.32^c^ (−6.21)	1135 ± 40.24^a^(+ 35.52)	989 ± 35.07^b^ (+ 18.09)
ALP (U/mg protein)	386 ± 14.72^a^	417 ± 15.17^a^ (+ 7.90)	262 ± 7.41^c^ (−32.12)	329 ± 12.50^c^ (−14.85)
ACP (U/L)	10.37 ± 0.273^a^	9.85 ± 0.351^a^ (−4.95)	6.25 ± 0.233^c^ (−39.71)	8.39 ± 0.212^b^ (−19.07)
Protein content (mg/g tissue)	72.55 ± 2.49^a^	76.57 ± 2.23^a^ (+ 5.55)	49.09 ± 1.50^c^(−32.33)	61.17 ± 1.93^b^ (−15.68)

Results are presented as means ± SE (*n* = 6/group). Various superscript letters (^abcd^) were used in each row to convey significance (*P* < 0.05) across groups. ω3FA and Insec Mix groups are contrasted to the negative control group, while the ω3FA plus Insec Mix group is related to the positive control group, Insec Mix.

### Hormonal levels and sperm quality

The present findings demonstrated that rats intoxicated with an insecticide mixture had decreased (*P* < 0.05) testosterone concentrations in their serum and increased LH and FSH levels compared to the control. Rats given ω3FA alone did not show a significant change in the examined hormones. In contrast to the insecticide-treated group, the concurrent intake of ω3FA and insecticide mixture caused these hormonal levels to be reversed. Data on sperm quality showed that rats treated with the insecticide mixture had considerably (*P* < 0.05) lower sperm count, motility, viability, and high sperm abnormalities than the control group. The assessed parameters exhibited minor modifications in rats treated with ω3FA alone. The co-administration of ω3FA + Insec Mix caused a significant recovery in sperm quality related to the Insec Mix-treated rats (Table 3).

**Table 3:** Effect of omega-3 fatty acid (ω3FA), insecticides mixture (Insec Mix), and their combination (ω3FA + Insec Mix) on hormone levels and sperm quality.

Parameters	Groups			
	Cont.	ω3FA	Insec Mix	ω3FA + Insec Mix
Testosterone (ng/ml)	3.16 ^a^ ±0.066	3.34 ^a^ ±0.125 (+ 5.70%)	0.77 ^c^ ±0.028 (−75.63)	2.08 ^b^ ±0.067 (−34.18)
FSH (ng/ml)	2.53 ^c^ ±0.091	2.46 ^c^ ±0.087 (−2.77)	3.92 ± 0.137^a^ (+ 54.94)	3.09 ± 0.055^b^ (+ 22.13)
LH (ng/ml)	2.85 ± 0.063^c^	2.65 ± 0.038^c^ (−7.02)	4.52 ± 0.166^a^ (+ 58.60)	3.38 ± 0.130^b^ (+ 18.60)
Sperm count (10^6^ Cells)	81.83 ± 3.36^a^	84.00 ± 2.51^a^ (+ 2.65)	20.50 ± 0.71^c^ (−74.95)	52.17 ± 1.11^b^ (−36.25)
Sperm motility %	79.17 ± 1.919^a^	81.00 ± 1.897^a^ (+ 2.31)	21.67 ± 0.704^c^ (−72.63)	55.17 ± 0.502^b^ (−30.32)
Abnormal sperm %	9.67 ± 0.195^c^	10.11 ± 0.294^c^ (+ 4.55)	17.17 ± 0.651^a^ (+ 77.55)	12.83 ± 0.442^b^ (+ 32.68)
Viability %	58.33 ± 2.11^b^	74.33 ± 0.95^a^ (+ 27.43)	22.33 ± 0.67^d^ (−61.72)	42.00 ± 1.29^c^ (−28.00)

Results are presented as means ± SE (*n* = 6/group). Various superscript letters (^abcd^) were used in each row to convey significance (*P* < 0.05) across groups. ω3FA and Insec Mix groups are contrasted to the negative control group, while the ω3FA plus Insec Mix group is related to the positive control group; Insec Mix.

### Gene expression

**Fig. 3:**
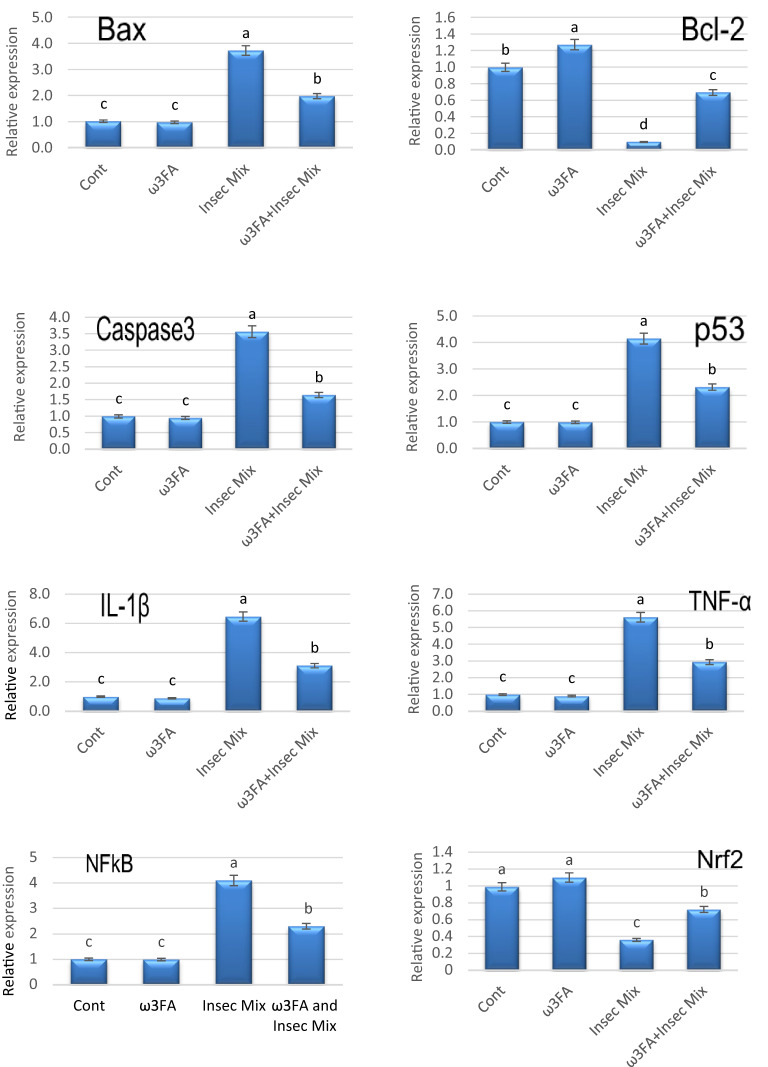
Apoptotic and inflammation-related genes in rats**’** testis tissues of different groups. Results are presented as means ± SE (*n* = 6/group). Various letters (a, b,c, and d) were used to convey significance (*P* < 0.05) across groups. ω3FA and Insec Mix groups are contrasted to the negative control group, while the ω3FA plus Insec Mix group is related to the positive control group, Insec Mix.

The qPCR results displayed a substantial (*P* ≤ 0.05) elevation in NFkB, Bax, Caspase3, P53, IL-1β, and TNF-α, and a decline in Nrf2 and Bcl-2 gene expression levels in the testis tissue of rats administered Insec Mix compared to control. The intake of ω3FA followed by Insec Mix treatment showed significant downregulation of NFkB, Bax, Caspase3, P53, IL-1β, and TNF-α, and upregulation of Nrf2 and Bcl-2 gene expression related to the Insec Mix group. However, rats treated with ω3FA alone had insignificant effects on all the studied gene expressions, except Bcl-2 was upregulated significantly compared to the control group (Fig. 3).

### Histopathological findings

**Fig. 4:**
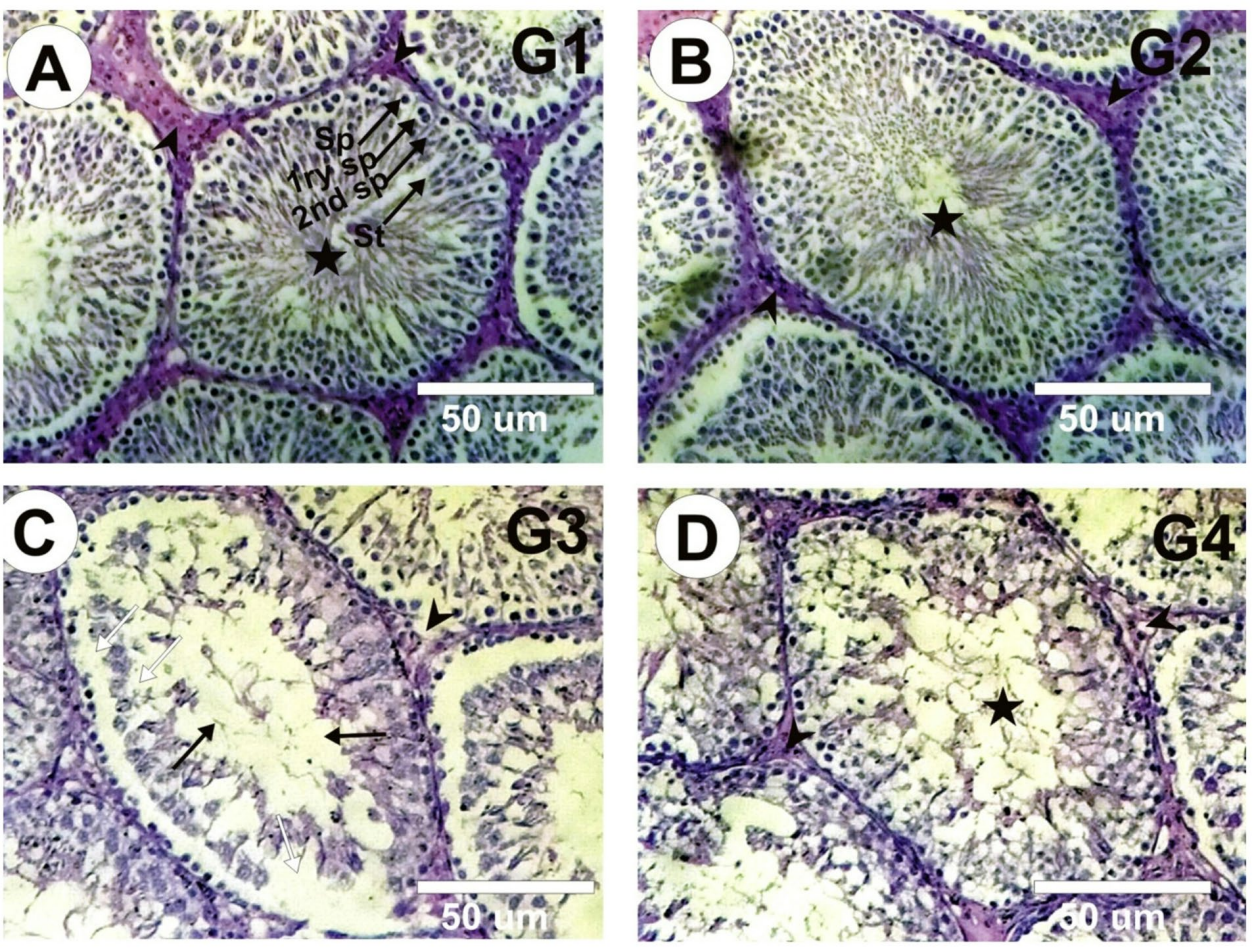
Hematoxylin and Eosin-stained photomicrographs of testes sections from the various experimental groups. (**A&B)** Normal structure of seminiferous tubules (arrows) and interstitial tissues (arrow heads). The cycle of spermatogenesis was regular, and the lumen of seminiferous tubules was fully packed with sperms (stars) in the control (G1) and ω3FA (G2) groups. (**C)** Rats treated with insecticide mixture (G3) revealed complete maturation arrest, abnormal arrangement of spermatogenesis cycles, severe noticeable degeneration (White arrows) in the majority of the seminiferous tubules (Black arrows), disturbance, and sloughing of germ cells into the tubular lumen with reduction of sperm numbers (Black arrows). (**D)** Testis section in rats co-treated with ω3FA and insecticide mixture (G4) showed good amendment with entire spermatogenesis, and a notable rise in sperm numbers (star).

Testicular sections of control (G1) and ω3FA-treated rats (G2) demonstrated a regular spermatogenesis cycle and a normal seminiferous structure. Conversely, testicular sections of insecticide-intoxicated rats (G3) displayed severe morphological changes, including abnormal arrangement of spermatogenesis cycles, a marked decline in the spermatogenic cells and sperm number in the seminiferous tubules, and moderately atrophied seminiferous tubules with degenerative Sertoli cells. Testicular sections of animals treated with both ω3FA and Insect Mix (G4) displayed a considerable change in sperm counts, mild atrophied seminiferous tubules, mild degeneration, and a satisfactory improvement with complete spermatogenesis (Fig. 4). Evidenced by a distorted histoarchitecture, scant sperm cells in the seminiferous tubule’s lumen, reduced Sertoli cells, and Leydig cell mass in rats treated with insecticides compared with the control. This was accompanied by an increase in testicular histoarchitecture and seminiferous luminal diameter and a decrease in epithelial height and seminiferous tubular diameter (Table 4).

**Table 4:** Effect of omega-3fatty acid (ω3FA), insecticides mixture (Insec Mix), and their combination (ω3FA + Insec Mix) on testicular cytoarchitecture.

Parameters	Groups			
	Cont.	ω3FA	Insec Mix	ω3FA + Insec Mix
Testicular histoarchitecture	1.33 ± 0.21^b^	1.40 ± 0.20^b^	4.50 ± 0.14^a^	2.13 ± 0.11^c^
Epithelial Height (µm)	68.91 ± 3.86^a^	70.01 ± 2.14^a^	40.08 ± 2.01^c^	59.04 ± 3.01^b^
Seminiferous Tubular Diameter (µm)	325 ± 10.39^a^	327 ± 13.54^a^	183 ± 7.35^c^	260 ± 5.32^b^
Seminiferous Luminal Diameter (µm)	38.02 ± 5.10^c^	40.36 ± 3.51^c^	143.5 ± 5.44^a^	81.62 ± 3.558^b^

Results are presented as means ± SE (*n* = 6/group). Various superscript letters (a, b,c, and d) were used in each row to convey significance (*P* < 0.05) across groups. ω3FA and Insec Mix groups are contrasted to the negative control group, while the ω3FA plus Insec Mix group is related to the positive control group, Insec Mix.

### KI67 immunohistochemical changes

**Fig. 5:**
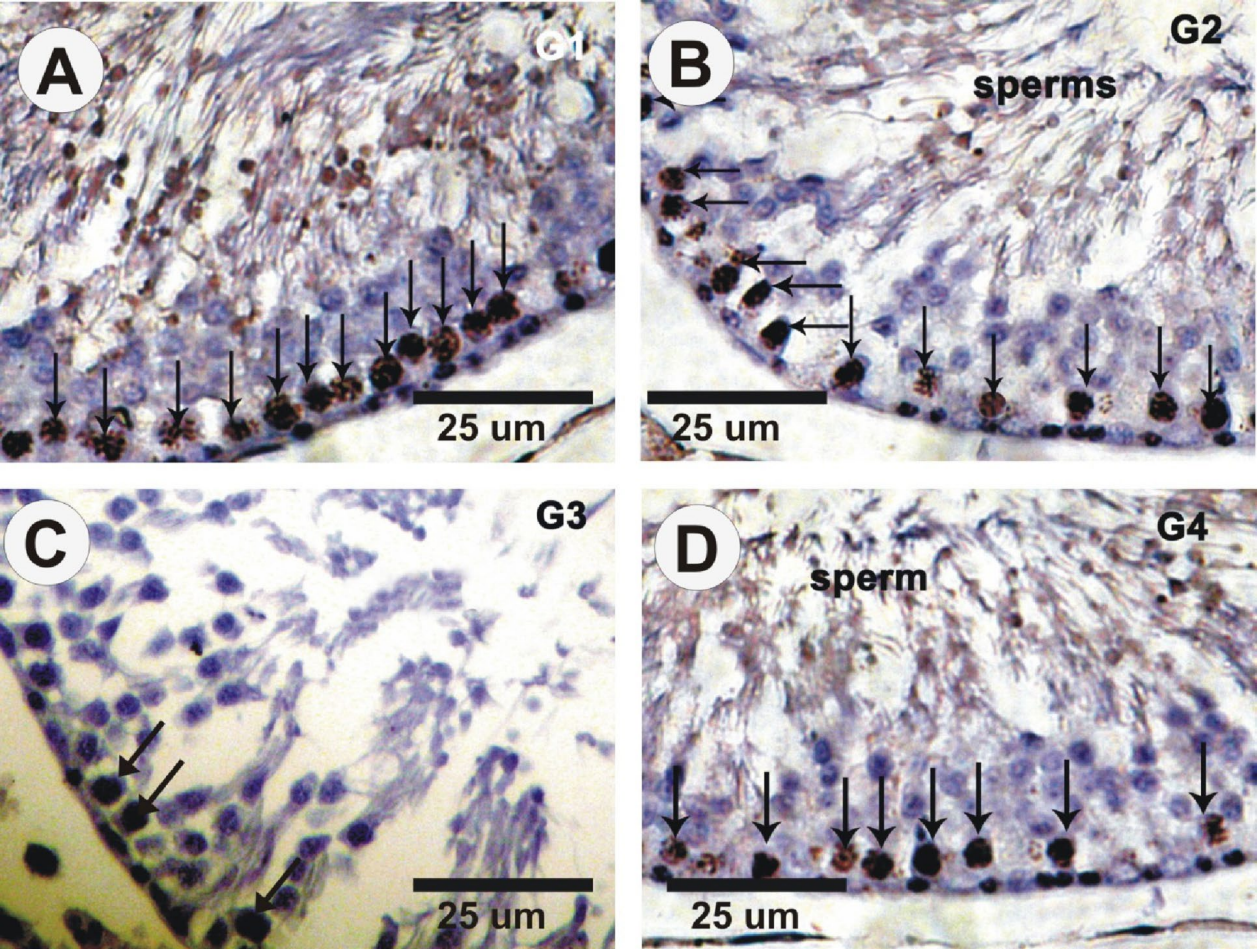
Photomicrographs of testis sections stained with Ki67 in different groups. (**A&B)** Strong affinity (arrows) in control (G1) and ω3FA (G2) groups. (**C)** Mild positive affinity for KI67 (arrows) in rats treated with pesticides (G3**)**. (**D)** Moderate positive affinity for KI67 (arrows) in rats administered ω3FA and insecticides (G4**)**.

The distribution and detection of KI67 immunoreactivity (KI67-ir) on testicular sections in control (G1) and ω3FA (G2) groups offered an intense “positive affinity for Ki67” in “spermatogonia”. However, rats treated with Insect Mix (G3) revealed mild positive affinity for KI67” in spermatogonia, while a moderate “positive affinity for KI67” was detected in rats (G4) given ω3FA and Insect Mix (Fig. 5).

## Discussion

The extensive use of pesticides exposes nontarget animals to pesticide residues in food and the environment regularly. Although acetamiprid and emamectin benzoate are generally accepted to be safe for the environment, they may pose a serious risk to nontarget creatures, such as aquatic invertebrates and animals^35^. As far as we know, insufficient information exists about the effectiveness of ω3FA in mitigating the toxicity of the insecticide mixture, acetamiprid, and emamectin benzoate on testicles. Therefore, the current study examined how ω3FA protects against DNA damage, alteration of ROS homeostasis, and testicular dysfunction caused by insecticides. Rats exposed to insecticides showed an increase in the oxidative stress indicators, TBARS and H_2_O_2,_ suggesting that biological membranes have undergone lipid peroxidation, changing their structure and function, decreasing their fluidity, and rendering several membrane-bound enzymes inactive^36^. Furthermore, several studies have reported that exposure to EMB in rats and mice’s brain, liver, kidney, and testis displayed high levels of lipid peroxidation byproducts and low GSH content, SOD, GPx, and CAT enzymes activity^37,38^. Also, acetamiprid increased the levels of malondialdehyde (MDA) and nitric oxide (NO) and decreased CAT activity in rat testes, which may have exacerbated oxidative stress^39^.

Since the antioxidant defense system is crucial for preserving the equilibrium of cellular function, it shields cells from oxidative damage induced by xenobiotic^40^. In line, Jebur et al.^4^ and Rahib et al.^41^ found that GSH content decreased in rats subjected to Fenpropathrin and abamectin, respectively. GSH, a non-enzymatic radical scavenger, is part of the second line of defense. It scavenges leftover free radicals from oxidative metabolism that avoid being broken down by antioxidant enzymes^42^. GSH redox cycles are crucial for cellular antioxidant defenses because they enable tissues to defend against ROS damage. They help remove ROS by acting as a substrate for many enzymes, including GPx, and as a non-enzymatic antioxidant and scavenger of oxygen radicals^43^. GSH can be generated through a reaction that is catalyzed by GR and transforms GSSG into GSH, or it can be produced spontaneously, with growth factors, oxidants, and antioxidants, regulating the process^44,45^. In line with our results, Temiz^38^ and Mahmoud et al.^46^ discovered that biopesticides emamectin benzoate and abamectin significantly increased oxidative toxicity as indicated by decreased GSH and elevated TBARS levels.

Depletion of the enzyme’s substrates or direct effects of the insecticide mixture on the enzyme could be the cause of the observed drop in GSH content and antioxidant enzyme activity^19,37^. Furthermore, two important antioxidant enzymes that protect biological macromolecules from oxidative damage are superoxide dismutase and catalase. SOD transforms superoxide anion radicals into hydrogen peroxide, a hazardous metabolic process, while CAT reacts with hydrogen peroxide to turn it into water. Consequently, cell death and deterioration of proteins and DNA could arise from the suppression of CAT activity^36^. Furthermore, to delay the conversion of GSSG to its reduced form through GR deactivation, insecticides may also impact GSH biosynthesis by inhibiting glutathione-synthase activity and decreasing NADPH, provided by glucose 6-phosphate dehydrogenase^36^. Additionally, GPx safeguards the cell membrane lipids against oxidative damage by initiating the interaction between hydroperoxide and GSH to create GSSG^47^. Furthermore, GST is crucial in detoxification and defense mechanisms against free radicals and electrophiles^48^. The findings also corroborate those of Jebur et al.^4^ and Ranjbar et al.^49^, who found that GST is an important enzyme engaged in the organophosphorus and pyrethroid insecticides’ quick binding and turnover or detoxification to non-toxic chemicals. Several authors have also noted that pesticide exposure reduces enzymatic antioxidants^2,50^, revealing that the antioxidant defense system can’t overcome the ROS influx produced by insecticides.

The reported reduction in aminotransferases in the testes homogenate of rats poisoned with insecticides might be due to damaged cells, modifications in the permeability of cell membranes, and protein metabolism^1,2,4^. Phosphatases are essential for metabolic processes, toxin scrapping, and the biosynthesis of energy macromolecules (ALP, ACP). The enzyme acid phosphatase is necessary for the enlargement of blood capillaries between seminiferous tubules, cell metabolism, breakdown, and differentiation^51^. The reduced ALP activity in the testes might be due to cell necrosis, allowing enzymes to leak into the bloodstream^52^. Rats treated with insecticides may exhibit elevated LDH activity due to significant cellular damage and impaired protein and carbohydrate metabolism, which are signs of metabolic abnormalities and a definite reaction to energy depletion^53^. Additionally, the reduction of cellular activity and disruption in protein synthesis are linked to the reported decrease in protein content in the homogenate of testes^4,54^. Furthermore, El-Sheikh and Galal^37^ reported that emamectin benzoate changed the liver’s levels of MDA and the activities of ALT, ALP, and SOD. Moreover, acetamiprid was discovered to disrupt the male mouse reproductive system, according to Zhang et al.^55^. This further demonstrated that acetamiprid’s metabolites were the source of its negative effects.

Environmental pollutants are known to affect hormones and change the function of the testicles^56^. According to Mathur and D’Cruz^57^, exposure to these pollutants has been associated with a decline in male fertility as indicated by a disturbance in sperm quality because they can imitate naturalistic estrogens and impact spermatogenesis, steroidogenesis, and the activity of Leydig and Sertoli cells. The generation of androgens and spermatozoa and the initiation of male reproductive function largely depend on hormones. Endocrine, paracrine, and autocrine processes are included in the interactions with the hypothalamic-pituitary-gonadal axis^58^. The decline in testosterone concentration suggests that the Leydig cells are not producing as much testosterone. According to El-Demerdash et al.^54^, increased LH levels are indications of Leydig cell dysfunction and a decline in endocrine function. The current study’s observation of an elevated FSH level suggests that the germinal epithelium is dysfunctional. In addition, the higher LH and FSH levels observed are consistent with Zhang et al.^55^, who discovered that acetamiprid dramatically lowered the rate of intact acrosomes, sperm motility, viability, and serum testosterone concentration. Additionally, acetamiprid produced problems in the Leydig cells’ mitochondria and endoplasmic reticulum, as well as harm to the seminiferous tubules and the process of spermatogenesis.The generation of ROS has been linked to infertility, deterioration in sperm quality, and reduction in sperm-oocyte fusion potential. Excess ROS harms spermatozoa with high polyunsaturated fatty acids content^59,60^. Rats exposed to insecticides produced less androgen or exhibited higher levels of testicular lipid peroxidation, explaining the noted drop in sperm count and motility and the increase in sperm morphological defects^61^.

The proportion of pro- and anti-apoptotic proteins in the Bcl-2 family affects cell life and death. Bcl-2 may act as a buffer to minimize injury by reducing lipid peroxidation caused by cytotoxic stressors such as ROS^62^. Additionally, it was discovered that Bcl-2 prevented cytochrome c from being released. However, Bax regulates apoptosis by dimerizing with Bcl-2 anti-apoptotic proteins, which release cytochrome c, and subsequently activate caspase-3^63^. In the present investigation, insecticide treatment markedly elevated the expression of Caspase-3, P53, and IL1β genes in the rats’ testes. In a related study, Temiz^38^ discovered that the toxicity of the biopesticide emamectin benzoate resulted in genotoxicity and an increase in the levels of the DNA oxidation biomarker 8-OHdG, along with its impact on lipid, protein, DNA, cellular macromolecules, and cell defense mechanisms. Also, after acute inflammation, monocytes and macrophages release the inflammatory cytokine TNF-α because it sets off a variety of signaling cascades in cells that cause necrosis or apoptosis. In addition, histological analysis of the testes of rats given insecticides showed several anomalies due to oxidative damage that may have accelerated the death of germ cells, decreased sperm quantity, and compromised the gonads’ integrity and activity^4,64,65^.

The combined toxicity of acetamiprid and emamectin benzoate in male reproductive tissues is not a simple additive effect but a synergistic cascade that disrupts fundamental cellular processes. Recent discoveries, as detailed in the papers, outlined a clear pathological pathway: The primary insult is a massive generation of ROS. This is consistently shown across all studies by elevated Malondialdehyde (MDA), a key marker of lipid peroxidation^6,8^. This occurs because the pesticides overwhelm the testicular antioxidant defense system, depleting enzymatic and non-enzymatic antioxidants^6,66^. The testes are particularly vulnerable due to their high concentration of polyunsaturated fatty acids in the membranes of sperm cells. Furthermore, oxidative stress triggers two critical signaling pathways as Nrf2, which is the master regulator of antioxidant response. Under oxidative stress, its activity is suppressed, preventing the cell from activating its own defense genes. This leaves the tissue defenseless against further damage. Also, ROS activates the NF-κB pathway, a primary regulator of inflammation. This leads to the increased production of pro-inflammatory cytokines such as Tumor Necrosis Factor-alpha (TNF-α) and Interleukin-1 beta (IL-1β), creating a state of chronic inflammation within the testicular tissue. The inflammatory and oxidative milieu pushes germ cells toward apoptosis. This is mediated by an increase in p53 (a tumor suppressor gene that arrests the cell cycle and promotes apoptosis) and Bax (a pro-apoptotic protein), and a decrease in Bcl-2 (a protein that promotes cell survival). This imbalance leads to mitochondrial dysfunction and the activation of Caspase-3, the executioner enzyme that systematically dismantles the cell^6,10^.

The synthesis of the protein Ki-67 is only linked to cell proliferation^34^. While the antigen is only found inside the nucleus during interphase, the majority of the protein is moved to the chromosomal surface during mitosis. The Ki-67 protein is a helpful indicator for figuring out the so-called proliferation of a specific cell population, as it is absent in resting cells but present in all active phases of the cell cycle. The protective influence of ω3FA in testicular dysfunction caused by pesticide mixtures is another significant discovery from this investigation. The current research showed that ω3FA lowered the level of lipid peroxidation in testis tissue. In agreement, Sarsılmaz et al.^67^ found that rats fed ω3FA had much lower TBARS values and that the antioxidant system in rodents was positively affected. Also, Eraky and Abo El-Magd^68^ reported that the pretreatment of ω3FA demonstrated antioxidant characteristics, as shown by a drop in MDA levels and an elevation in total antioxidant capacity alongside improved liver functions. Strong antioxidant properties of ω3FA seemed to dramatically counteract oxidative damage caused by insecticides by improving sperm quality, including sperm motility and count.

Furthermore, it has been established that ω3FA promotes anti-apoptotic signaling, eliminates oxygen and nitrogen-based reactants generated in the mitochondria, and maintains the mitochondrial membrane^31^. The decreased levels of inflammatory factors in the treatment groups may be due to ω3FA’s antioxidant properties and ability to block TNF-α production. According to a related study by Odetayo et al.^32,33^, ω3FA enhanced sperm quality, reproductive hormone synthesis, testicular structure, and increased Ki-67 expression by inhibiting signs of testicular injury, oxidative stress, inflammatory response, and apoptotic markers. For the first time, our study demonstrates that ω3FA reduces testicular dysfunction caused by insecticide mixtures. These results were consistent with earlier research that documented ω3FA’s anti-inflammatory^69^, anti-apoptotic^70^, and antioxidant^71^ properties. Thus, our work demonstrates that ω3FA enhanced testicular functioning in rats treated with insecticide mixtures by maintaining testicular redox balance and boosting sperm quality and reproductive hormones.

Nuclear Factor Erythroid Related Factor 2 (Nrf2) and Nuclear Factor-Kappa B (NFkB) are key regulators of the body’s response to oxidative stress and inflammatory response^72^. During excessive and continuous exposure to external stress, the body produces excess free radicals and ROS, leading to the downregulation of endogenous antioxidants, thereby damaging the body’s cellular components such as proteins, DNA, and lipids^73^. Nrf2 is a major endogenous antioxidant controlling various aspects of cellular homeostasis in response to oxidative stress^74^. The decline in Nrf2 due to external stressors can upregulate NFkB expression, leading to an inflammatory response. Also, the increase in NFkB expression can lead to a further decrease in Nrf2. Hence, Nrf2 and NFkB are important players in the crosstalk between oxidative stress and inflammation^75^. The excessive decrease in the endogenous antioxidant system and increased inflammatory response can trigger an apoptotic response^28^. On the other hand, supplementation of exogenous antioxidants can target oxidative stress by inhibiting the production of free radicals and ROS and bolstering the endogenous antioxidant capacity.

Although this study demonstrates for the first time that ω3FAs ameliorate acetamiprid and emamectin benzoate-induced testicular dysfunction, these findings concurred with previous findings that reported the antioxidant^71^, anti-inflammatory^69^, and antiapoptotic^70^ activities of ω3FAs. The observed redox balance and downregulation of NF-kB expression in the testicular tissues of ω3FA-treated animals possibly explained ω3FA’s driven repression of apoptotic markers via the upregulation of Nrf2 activities. The gonadoprotective effect of ω3FAs was accompanied by the restoration of testicular histoarchitecture and function by preventing distortion of histoarchitecture, scanty sperm cells in the lumen of the seminiferous tubule, reduced Leydig cell mass, and normalization of sperm qualities and reproductive hormones^6^.

Moreover, ω3FAs act as a multi-target therapeutic agent, countering pesticide-induced damage. Their protective role is not merely antioxidant but also anti-inflammatory and anti-apoptotic. ω3FAs are incorporated into cell membranes, making them more resilient to oxidative attack. More importantly, they upregulate the Nrf2 pathway. By enhancing Nrf2 signaling, ω3FAs boost the cell’s own production of a wide array of antioxidant enzymes, creating a sustained defensive state against ROS^14^. ω3FAs (specifically EPA and DHA) are precursors to specialized pro-resolving mediators (SPMs) like resolvins and protectins, which are powerful endogenous anti-inflammatory molecules. They directly inhibit the NF-κB pathway, reducing the production of pro-inflammatory cytokines (TNF-α, IL-1β). This quells the inflammation within the testicular tissue^16^. By reducing oxidative stress and inflammation, ω3FAs remove the primary triggers for apoptosis. They directly modulate the apoptotic machinery by downregulating Bax and p53 and upregulating Bcl-2, thereby shifting the balance away from cell death and toward cell survival^17,33^.

Also, Ki-67 serves as a marker of cell proliferation and plays a crucial role in spermatogenesis within the testes. Its expression is commonly used to assess male reproductive maturity, testicular damage or toxicity, and overall fertility potential. Testicular cancer (such as seminomas), degenerative testicular illnesses, and hormonal or chemical damage to the testes can all be diagnosed and tracked with the use of Ki-67 expression. While low levels may indicate testicular failure, high levels of Ki-67 expression are indicative of an active spermatogenic cycle, healthy seminiferous tubules, and good reproductive function^34^. In the present study, rats treated with Insect Mix showed a significant decrease in Ki67 expression, indicating testicular dysfunction, injury, and toxicity, whereas rats treated with Insect Mix and ω3FA showed an increase in Ki67 expression, indicating the activation of the spermatogenic cycle.

## Conclusion

In conclusion, the present finding demonstrated that ω3FA pre-treatment improved antioxidant status and reduced oxidative stress, inflammation, hormonal imbalance, poor sperm quality, and apoptosis in rats treated with insecticides by modulating gene expression. These results raise the possibility of understanding the molecular pathways by which ω3FA protects against testicular dysfunction caused by insecticides.

## Data Availability

The data sets used and/or analyzed during the current study are available from the corresponding authors upon reasonable request.

## Abbreviations

ROS: Reactive oxygen species
DHA: Docosahexaenoic acid
EPA: Eicosapentaenoic acid
ω3FA: Omega-3fatty acids
ACP: Acetamiprid
EMB: Emamectin benzoate
TBARS: Thiobarbituric acid reactive substances
H_2_O_2_: Hydrogen peroxide
SOD: Superoxide dismutase
CAT: Catalase
GPx: Glutathione peroxidase
GR: Glutathione reductase
GST: Glutathione S-transferase
GSH: Reduced glutathione
AST: Aspartate aminotransferase
ALT: Alanine aminotransferase
LDH: Lactate dehydrogenase
ALP: Alkaline phosphatase
AcP: Acid phosphatase
T: Testosterone
LH: Luteinizing hormone
FSH: Follicle-stimulating hormone
GAPDH: Glyceraldehyde-3-phosphate dehydrogenase
ABC: Avidin-Biotin-Peroxidase
MDA: Malondialdehyde
NO: Nitric oxide
Bcl-2: B-cell lymphoma-2
Bax: Bcl2-associated X protein
TNF-α: Tumor necrosis factor-alpha
IL1β: Interleukin- 1beta
